# The effect of Enright Process and REACH forgiveness models on promoting forgiveness among college students in Ashanti, Ghana

**DOI:** 10.3389/fpsyg.2026.1780264

**Published:** 2026-04-10

**Authors:** Grace Aba Mensah, Godwin Awabil, Grace Yeboah, Eric Atta Quainoo, Kennedy Nyeseh Ofori

**Affiliations:** 1 Department of Education, Wesley College of Education, Kumasi, Ghana; 2 Counselling Centre, University of Cape Coast, Cape Coast, Ghana; 3 Department of Interdisciplinary Studies, AAMUSTED, Kumasi, Ghana

**Keywords:** College of Education, Enright Process, forgiveness, REACH models, students

## Abstract

**Introduction:**

Forgiveness interventions have gained increasing attention in counselling psychology as effective approaches for addressing interpersonal hurt among students. However, empirical evidence comparing the effectiveness of the Enright Process Model and the REACH Forgiveness Model within the Ghanaian college context remains limited. This study therefore explored the effects of these two models on forgiveness among college education students in the Ashanti Region of Ghana.

**Methods:**

The study employed a quasi-experimental pre-test, post-test control group design. The population comprised second-year students from three selected Colleges of Education in the Ashanti Region. A sample of 60 students who met eligibility criteria and reported experiencing interpersonal hurt was selected and randomly assigned into three groups: two experimental groups (Enright Process and REACH models) and one control group, each consisting of 20 participants (10 males and 10 females). Data were collected using the Enright Forgiveness Inventory (EFI) and a semi-structured interview guide. Quantitative data were analyzed using one-way and two-way analysis of covariance (ANCOVA), while qualitative data were analyzed thematically.

**Results:**

The findings revealed that both the Enright Process Model and the REACH Forgiveness Model significantly enhanced participants’ levels of forgiveness compared to the control group. The ANCOVA results indicated statistically significant differences in post-test forgiveness scores among the groups, with participants in both intervention groups demonstrating notable improvements. Thematic analysis of interview data supported these findings, highlighting increased emotional healing, empathy, and reduced resentment among participants.

**Discussion:**

The study demonstrates that both the Enright Process and REACH models are effective interventions for promoting forgiveness among college students experiencing interpersonal hurt. These findings underscore the relevance of structured forgiveness therapies in educational and counselling settings in Ghana.

**Recommendation:**

It is recommended that counsellors integrate these models into intervention programs to enhance students’ emotional well-being and interpersonal relationships.

## Introduction

Interpersonal conflicts are a normal component of human relationships and daily life. People may feel offended or upset by others’ conduct, which can range from simple misunderstandings to significant acts of violence. These interpersonal transgressions can take various forms, including bullying, child abuse, elder abuse, and intimate partner violence, all of which can have severe emotional and psychological implications for victims. Conflicts and misbehavior are common in close relationships due to differing attitudes, interests, values, and behaviors. As a result, interpersonal disagreements and violations typically elicit negative emotional responses such as anger, resentment, and hostility, endangering the stability and quality of relationships (Fincham et al., 2002; Krug et al., 2002).

When negative emotions like resentment and anger are suppressed or maintained over time, they can lead to psychological and physical health issues. Chronic anger and unresolved interpersonal conflict have been linked to higher levels of stress, depression, and other health problems (Chida and Steptoe, 2009; Goldman and Wade, 2012). In response to these issues, therapists and researchers have stressed the importance of forgiveness as a coping strategy for dealing with interpersonal conflicts. Forgiveness has been linked to lower negative emotional states and higher psychological wellbeing (Worthington and Scherer, 2004). Forgiving those who inflict harm is regarded as vital in many cultures for reconciliation, healing, and peacebuilding (Nyarko and Punamäki, 2017). In contrast, the inability to forgive has been associated with negative psychological effects such as sadness and emotional discomfort (Maltby et al., 2001).

Forgiveness is typically regarded as a complex cognitive, emotional, and behavioral process in which people diminish their feelings of resentment and embrace more positive views toward the offender (Worthington, 2005). It entails replacing negative feelings like anger, vengeance, and bitterness with compassion, empathy, or understanding for the transgressor. Forgiveness is thus seen as a key tool for repairing damaged relationships and facilitating emotional recovery. According to scholars, forgiveness can help avert future conflicts, improve reconciliation between individuals or groups, and promote peaceful cohabitation within communities (Worthington et al., 2019). Research indicates that those who practice forgiveness have lower levels of rumination, animosity, and self-centeredness, and are also more empathetic and emotionally resilient than those who do not forgive (Worthington, 2006).

Over time, numerous theoretical models have emerged to guide forgiveness therapies and therapeutic practices. Two of the most widely recognized models are the Enright Process Model of Forgiveness and Worthington’s REACH Forgiveness Model. The Enright Process Model views forgiveness as a gradual process that involves emotional, cognitive, and behavioral transformation in those who have experienced interpersonal injury. This methodology stresses incremental changes in thoughts, emotions, and actions toward the offender through a structured set of steps that assist people to recognize their pain and gradually develop more compassionate responses (Ingersoll-Dyaton et al., 2008). The process often begins with minor behavioral changes, such as refraining from making disparaging remarks about the offender, and then leads to deeper emotional and cognitive reconciliation.

Similarly, Worthington (2001) created the REACH Forgiveness Model, a psychoeducational and cognitive–behavioral strategy for facilitating forgiveness through structured therapeutic procedures. REACH is an acronym that represents five steps: Recall the harm (R), empathize with the perpetrator (E), give an altruistic gift of forgiveness (A), commit to forgive (C), and maintain forgiveness (H). Individuals engage in cognitive and emotional reflection during these processes, allowing them to process traumatic experiences and develop a more compassionate attitude toward offenders. Research has shown that the REACH model can effectively reduce unforgiveness, promote empathy, and improve participants’ emotional wellbeing (Wade et al., 2014; Worthington, 2020). The concept is also adaptable and has been culturally modified in various contexts, with studies indicating that culturally tailored REACH interventions can successfully enhance forgiveness and empathy across diverse groups (Toussaint et al., 2020; Kurniati et al., 2020). Empirical research, primarily conducted in Western contexts, has consistently demonstrated that structured forgiveness interventions, such as the Enright Process Model and the REACH Forgiveness Model, can significantly improve psychological wellbeing and reduce negative emotions associated with interpersonal offenses. These interventions have been used with a variety of populations, including adolescents, adults, couples, and survivors of interpersonal violence. Despite the growing body of evidence supporting forgiveness therapy globally, empirical research on these interventions in African contexts remains limited.

In Ghana, recent research has begun to investigate forgiveness interventions among students and counselor trainees (Akosah et al., 2025; Barimah, 2019; Kankpog et al., 2023). While these studies provide valuable insights into the efficacy of forgiveness therapy, the majority focus on specific intervention modalities or particular psychological outcomes. Furthermore, little research has been conducted on forgiveness interventions among College of Education students, who are prospective teachers expected to promote social harmony, emotional development, and conflict resolution in school settings. More importantly, few studies have directly compared the Enright Process Model with the REACH Forgiveness Model using the same experimental paradigm, particularly among Ghanaian teacher trainees. This study addresses this gap by examining and comparing the effects of the Enright Process Model and the REACH Forgiveness Model on forgiveness among students in Ghana’s Ashanti Region Colleges of Education, using a quasi-experimental design supplemented by qualitative insights from participants. The study examined the following hypotheses:

*Hypothesis 1:* Participants who receive the Enright Process Model and REACH Forgiveness Model intervention will demonstrate significantly higher forgiveness scores at post-test compared to participants in the control group.

*Hypothesis 2:* There will be a statistically significant difference in forgiveness scores between participants exposed to the intervention models and those in the control group after controlling for pre-test scores.

## Methodology

### Research design and approach

This study used a separate design and approach, combining quantitative and qualitative methods to provide a comprehensive understanding of the impact of forgiveness programs. The quantitative component was guided by the post-positivist paradigm, which emphasizes objective measurement, hypothesis testing, and empirical investigation of causal linkages. This paradigm was suited for the study since it used standardized questionnaires and statistical analysis to assess the impact of organized forgiveness programs on students’ levels of forgiveness.

The quantitative strand used a quasi-experimental, pre-test–post-test, non-equivalent control group design. This design was appropriate since participants were chosen from intact institutional groups in colleges of education, where random assignment of individuals to experimental and control groups was not possible. Quasi-experimental designs enable researchers to study the impact of interventions in natural settings while maintaining sufficient control over confounding variables. The design featured two experimental groups and one control group, which were structured as follows:

- Experimental Group A: Participants received the *Enright Process Model forgiveness intervention*
- Experimental Group B: Participants received the *REACH Forgiveness Model intervention*
- Control Group: Participants did not receive any intervention during the study period.

Participants in all groups completed a pre-test assessment of forgiveness, after which the experimental groups participated in an 8-week forgiveness intervention program while the control group received no treatment. After the intervention period, all participants completed a post-test. The intervention process was in line with the pre-test, post-test, control group design. Table 1 indicates the procedure.

**Table 1:** Pre-test, post-test, and control group design.

Group	Group A	Group B	Group C
Pre-test	O_1_	O_2_	O_3_
Treatment	X_1_	X_2_	
Post-test	O_4_	O_5_	O_6_

From Table 1, O_1_, O_2_, and O_3_ represent the pre-test, X_1_ and X_2_ represent the treatments that were carried out, and O_4_, O_5_, and O_6_ represent the post-test. As the design indicates, the experimental groups—specifically the Enright Process group (A), REACH group (B), and the control group (C)—were all pre-tested and post-tested. Only the experimental groups (A & B) received the treatment. Group C, the control group, did not receive any treatment. Control group members were allowed to continue their normal activities but also participated in the post-test. To avoid diffusion, the experimental and control groups were located in different settings.

To supplement the quantitative findings and gain deeper insights into participants’ experiences with the therapies, the study included a qualitative component based on a phenomenological design. The phenomenological approach was chosen because it focuses on investigating individuals’ actual experiences and personal perceptions of a phenomenon, in this case, forgiveness after the intervention. Following the intervention phase, semi-structured interviews were conducted with selected participants to learn about their experiences with the forgiveness process and the perceived impact of the intervention. By combining quantitative and qualitative methodologies, the study was able to quantify the statistical success of the interventions while also understanding participants’ subjective experiences, resulting in a more comprehensive interpretation of the results.

### Area of the study

The study was conducted in three selected colleges of education in Ghana’s Ashanti Region. The Ashanti Region was chosen because it has a large number of educational colleges and a significant population of Ghanaian teacher trainees. These institutions train prospective teachers to play crucial roles in promoting positive interpersonal interactions, emotional development, and conflict resolution among students in schools. The study was deemed essential since teacher trainees frequently confront interpersonal disputes in academic and social settings. Investigating forgiveness therapies in this context provides insights into strategies that may assist future educators in acquiring emotional control and conflict resolution skills applicable in educational settings.

### Population and sampling

The study’s target group included all second-year students enrolled in the designated Colleges of Education in Ghana’s Ashanti Region during the academic year 2024/2025. Second-year students were chosen because they had spent enough time in the college setting to experience interpersonal interactions and disagreements with peers.

A two-stage sampling approach was utilized. First, purposive sampling was utilized to identify students who had experienced interpersonal injury or disagreement and found it difficult to forgive. This ensured that the individuals were suitable for the forgiveness intervention. During the initial orientation session, students were asked if they had experienced interpersonal or emotional pain caused by another person and if they were willing to participate in a forgiveness-focused counseling program. The study’s 60 participants were chosen through simple random sampling from a pool of eligible students. Because the participants belonged to existing student groups within the colleges, random assignment to treatment conditions was not possible. Therefore, the selected participants were placed into three intact groups consisting of 20 participants each:

- Experimental Group A (Enright intervention) – 20 participants
- Experimental Group B (REACH intervention) – 20 participants
- Control Group – 20 participants

This grouping structure allowed the researchers to compare the effectiveness of the two forgiveness programs to a non-treatment group.

The Enright Forgiveness Inventory (EFI) was administered as a pre-test measure to assess participants’ baseline levels of forgiveness toward the identified offender. This process helped ensure that participants had unresolved feelings associated with the offense and were therefore suitable candidates for the forgiveness intervention. Through this screening process, the researchers ensured that participants were meaningfully engaged with the issue of forgiveness and that the intervention addressed real interpersonal experiences rather than hypothetical situations. See Table 2.

**Table 2:** A summary of the demographic information of participants.

	GROUPS							
	Enright Process		REACH		Control		Total	
	*N*	%	*N*	%	*N*	%	*N*	%
Gender								
Male	10	50.0	10	50.0	10	50.0	30	50.0
Female	10	50.0	10	50.0	10	50.0	30	50.0
Age (years)								
17–20	10	50.0	9	45.0	7	35.0	26	43.3
21–24	8	40.0	8	40.0	9	45.0	25	41.7
25 and above	2	10.0	3	15.0	4	20.0	9	15.0
Total	20	100	20	100	20	100	60	100.0

Out of the 60 participants, 50% were male and 50% were female. Each of the three groups (Enright Process group, REACH group, and control group) also had an equal gender distribution, with 50% male and 50% female. This balanced gender distribution across the groups was important for the study because it ensured that the intervention effects were not influenced by gender differences. The equal representation of male and female participants allowed the researchers to examine the effectiveness of the forgiveness interventions without bias toward a particular gender. As presented in Table 2, the majority of the participants were between the ages of 17 and 20 years (43.3%). This age group represents the typical range of students enrolled in Colleges of Education and reflects a developmental stage in which individuals are still developing emotional regulation and interpersonal relationship skills. The presence of a large proportion of participants within this age category suggests that many of the students may still be learning how to cope with interpersonal hurt and conflict, making them suitable candidates for forgiveness-based counseling interventions.

Half of the participants in the Enright Process group were within the age category of 17–20 years, and a similar pattern was observed in the REACH group, where the majority of participants were also in this age range. This similarity in age distribution between the two experimental groups was important because it ensured comparability between the intervention groups. In contrast, the control group had a slightly higher proportion of participants in the age range of 21–24 years. Although this variation exists, the overall age distribution across the groups remained relatively similar, suggesting that age differences were unlikely to significantly influence the outcomes of the intervention. Overall, the demographic characteristics of the participants indicate that the study sample consisted mainly of young adults who are likely to experience interpersonal conflicts within academic and social environments. This makes the population appropriate for examining the effectiveness of forgiveness-based counseling interventions. In their intact groups, the experimental groups A & B and the control group C were each handled by two research assistants under the supervision of the investigator. These research assistants, who were all professional counselors with data collection experience in the area of the research, underwent a 5-day training period, with each session lasting 2 hours before the pre-testing of the instruments. Although the two trained research assistants facilitated the intervention groups using standardized facilitator guides and participated in preparatory training and debriefing sessions, formal fidelity checks such as session-by-session checklists, independent fidelity ratings, and audio/video recordings were also systematically conducted. These measures ensured the consistency with which the intervention models were delivered across groups. In conducting the pre-test, 20 copies of the Attitude Scale of Robert Enright were administered to experimental groups A and B and the control group C.

Conducting the intervention, the two experimental groups A and B used the Enright Process model and the REACH model for the experimental treatment. The control group C was not given any treatment and was permitted to go about their normal daily activities. The experimental groups were taught by the researcher and the research assistants, who had been trained for 5 days on how to use the models. The interaction lasted for 2 hours per week over 8 weeks. This was in agreement with Lundahl et al.’ (2008) assertion that an effective forgiveness treatment must comprise a procedure that lasts for more than a day. Additionally, the two research assistants were allowed to handle at least two groups in each treatment condition.

Participants in experimental group A were exposed to the sources of their hurt, how to react to those hurts, the costs and benefits of committing to forgiveness, broadening their view of the person who hurt them, the nature of compassion, and working toward finding meaning in suffering. Participants in experimental group B were also exposed to the sources and concepts of forgiveness, recalling the hurt, empathizing with the one who hurt them, giving the altruistic gift of forgiveness, committing to forgiveness, and holding on to forgiveness. The interactions in the experimental groups (A and B) included discussions, role play, direct teaching instruction, reflections, and homework. Interactions were conducted in a friendly environment to ensure effective participation.

There was a 14-day interval before the post-test was conducted after 8 weeks of treatment. This was to ensure that participants did not replicate what they learned during the intervention period. Meanwhile, the instruments used for the pre-test were re-administered to the participants in both the treatment groups and the control group to acquire the post-test data.

After the quantitative data was collected and analyzed at the end of the intervention, qualitative data was also collected through interviews with six participants who were purposively selected to determine the effectiveness of the Enright Process model and the REACH model on forgiveness and depression. Before the interview, the interviewees were given copies of the interview schedule to study. This was done to facilitate communication between the interviewer and the interviewees.

### Instruments

#### Quantitative instrument

The study used the Enright Forgiveness Inventory (EFI) developed by Enright (2001) to measure participants’ levels of forgiveness. The EFI is a widely used self-report instrument designed to assess interpersonal forgiveness toward a specific offender. The inventory consists of 60 items divided into three primary subscales: affect (emotional responses toward the offender); behavior (behavioral intentions toward the offender); and cognition (thoughts and beliefs about the offender). These subscales assess six dimensions of forgiveness: absence of negative affect, presence of positive affect, absence of negative cognition, presence of positive cognition, absence of negative behavior, and presence of positive behavior toward the offender. Responses are rated on a six-point Likert scale ranging from 1 (Strongly Disagree) to 6 (Strongly Agree). Negative items are reverse scored. Total scores range from 60 to 360, with higher scores indicating higher levels of forgiveness. The EFI has demonstrated strong reliability and validity in previous studies. Reed and Enright (2006) reported a Cronbach’s alpha coefficient of 0.98, indicating high internal consistency.

#### Qualitative instrument

To obtain deeper insights into participants’ experiences of the forgiveness interventions, a semi-structured interview guide consisting of three open-ended questions was developed. The interview questions focused on participants’ experiences during the intervention, perceived changes in their emotions toward the offender, and how the intervention influenced their attitudes toward forgiveness. Interviews were conducted after the completion of the intervention program with selected participants from the experimental groups.

#### Validity and reliability of the instrument

The inventory was scrutinized by supervisors and other experts, such as counselors and peers, for discussions and comments. This aided in establishing the content validity of the inventory employed. The reliability estimates of the inventory ranged from 0.86 to 0.88 (Affect −0.86, Behavior −0.88, Cognition −0.88). From the viewpoint of Pallant (2005), reliability estimates above 0.70 are acceptable and sufficient to ensure reliability. Based on Pallant’s claim, all the reliability estimates of the scales employed in this research were adequate and consequently reliable. It was also necessary to establish the stability of the traits being measured. With respect to affect, a stability estimate of 0.864 (*p < 0*.001) was achieved, signifying that participants had consistent affect toward someone who had hurt them. The thoughts about a person who had hurt the participants (*Rt = 0.*897, *p < 0*.001), and the behavior toward a person who had hurt them (*Rt = 0*.889, *p < 0*.001) were all steady over the time period during which the administrations were conducted.

#### Intervention procedure

The intervention procedure was carried out in three phases, namely: pre-intervention phase, intervention phase, and post-intervention phase.

#### Pre-intervention phase

Before the intervention began, participants in all groups completed the Enright Forgiveness Inventory to assess their baseline levels of forgiveness toward the identified offender.

#### Intervention phase

Participants in the two experimental groups engaged in 8 weeks of structured counseling sessions based on the Enright Process Model and the REACH Forgiveness Model, respectively. Each session lasted approximately 60–90 min and included activities such as group discussions, reflective exercises, empathy development tasks, and guided counseling activities designed to promote forgiveness. Participants in the control group did not receive any intervention during this period.

#### Post-intervention phase

At the end of the intervention period, participants in all groups completed the Enright Forgiveness Inventory again to measure changes in forgiveness levels following the intervention.

### Data analysis

#### Quantitative data analysis

For quantitative data, a one-way analysis of covariance (ANCOVA) was employed to test the effects of the Enright Process and REACH models on forgiveness. This helped remove the impact of the pre-test from post-test performance. ANCOVA was carried out for each treatment group: the Enright Process model of forgiveness intervention and the REACH model of forgiveness intervention. The use of ANCOVA helped control for extraneous variables. The scores on the pre-test were treated as covariates to account for pre-existing differences between the groups. This aimed to eliminate the effect of participants’ exposure to the pre-intervention data collection instruments on the dependent variables (Pallant, 2005). ANCOVA achieves this by adjusting pre-test scores as a covariate to control for previous variances among groups.

The normality assumption was tested to determine whether a parametric test should be used. Emphasis was placed on the Shapiro–Wilk test because it is appropriate for data with a small sample size (Field, 2009). The results from the normality test revealed that the normality assumption was satisfied for both testing procedures. All the *p*-values for the variables were greater than 0.05 (i.e., level of significance), providing strong evidence for the non-violation of the normality assumption. According to the results from the Shapiro–Wilk test, forgiveness (pre-test) had a *p*-value of 0.298, and forgiveness (post-test) had a *p*-value of 0.270. Importantly, the homogeneity of regression slopes assumption was tested by including the interaction term between the covariate (pre-test scores) and the independent variable (group) in the model. The interaction was non-significant, *F*(1, 56) = 1.24, *p* = 0.27, suggesting that the relationship between the covariate and the dependent variable was consistent across groups. This confirmed that the assumption was satisfied. After meeting the normality assumption and the homogeneity of regression slopes assumption, an analysis of the responses was conducted.

#### Qualitative data analysis

Content analysis was used to analyze qualitative data to isolate it under specific content. First, the authors read the data repeatedly to become familiar with the depth and breadth of the data. This involved marking ideas and patterns for coding. The second phase involved generating initial codes by attaching names to pieces of text that related to specific research questions and the theoretical framework of the study. In the third phase, the codes were analyzed and sorted into potential themes. The potential themes were reviewed and refined in the fourth phase. In this phase, some of the themes were combined, and others were discarded. The fifth phase involved defining and naming themes by identifying the aspects of the data that each theme captures and how each theme relates to the research question(s).

### Ethical issues and data collection procedures

Before data collection began, ethical approval for the study was obtained from the Institutional Review Board (IRB) of the College of Education Studies at the University of Cape Coast. Several ethical measures were implemented to protect the rights and wellbeing of the participants throughout the study. Formal permission was first obtained from the principals of the selected Colleges of Education before the research was conducted. Participants were provided with detailed information about the purpose of the study, the procedures involved, potential risks, and their rights as participants, after which written informed consent was obtained. Participation in the study was entirely voluntary, and participants were informed that they could withdraw at any time without penalty. To ensure confidentiality and anonymity, identification codes were assigned to participants instead of using their names, and all collected data were securely stored and made accessible only to the research team. Because the intervention involved recalling interpersonal hurt, participants were informed about the emotional nature of the activities, and counseling support was made available for anyone who experienced emotional distress during the intervention. In addition, after the completion of the study, participants in the control group were provided with the forgiveness intervention materials so they could also benefit from the program. All questionnaires and interview recordings were securely stored, digital files were password-protected, and the data were retained strictly for academic purposes and destroyed after the required retention period. These measures ensured that the study adhered to ethical standards and safeguarded the dignity, safety, and psychological wellbeing of all participants throughout the research process.

## Results

*Hypothesis 1:* Participants who receive the Enright Process Model and REACH Forgiveness Model intervention will demonstrate significantly higher forgiveness scores at post-test compared to participants in the control group.

To test this hypothesis, a one-way analysis of covariance (ANCOVA) was conducted to examine the effect of the Enright Process Model and the REACH Forgiveness Model on participants’ post-test forgiveness scores while controlling for pre-test scores. The results of the ANCOVA are presented in Table 3.

**Table 3:** ANCOVA results on the effect of enright process model and REACH model on forgiveness.

Source	Type III Sum of squares	Df	Mean square	F	Sig.	Partial Eta squared
Corrected model	101144.84	3	33714.95	17.73	0.000	0.492
Intercept	61486.17	1	61486.17	32.33	0.000	0.370
Forgive (Pre-test)	364.31	1	364.314	0.19	0.663	0.003
Group	101118.18	2	50559.09	26.59	0.000	0.492
Error	104591.19	55	1901.66			
Total	3155825.00	59				
Corrected total	205736.034	58				

Dependent variable: forgiveness post-test.

The result, as shown in Table 3, revealed that after controlling for the pre-test scores of respondents on forgiveness, there was a statistically significant difference in the post-test forgiveness scores for the participants in the experimental groups and the control group, *F* (2, 55) = 26.59, *p* < 0.001. The result further indicated that the groups (Process model, REACH model, and control group) explained about 49.2% of the variations in forgiveness (η_p_2 = 0.492). According to Cohen (1988) guidelines, values of 0.01, 0.06, and 0.14 correspond to small, medium, and large effects, respectively. Thus, an effect size of 0.492 represents a very large effect, indicating that nearly half of the variance in post-test forgiveness scores can be attributed to the intervention after controlling for pre-test scores. Practically, this suggests that the intervention models (Enright Process and REACH) exerted a strong influence on participants’ forgiveness outcomes, producing meaningful and substantial improvement. The results necessitated a *post hoc* analysis to determine where the differences in marginal means scores exist among the groups. See Table 4.

**Table 4:** Sidak Adjustment for pairwise comparisons on forgiveness.

(I) Groups	(J) Groups	Mean difference (I-J)	Std. Error	Sig
REACH	Process	1.162	14.927	0.999
	Control	88.118^*^	14.140	0.000
Process	REACH	−1.162	14.927	0.999
	Control	86.956^*^	14.132	0.000
Control	REACH	−88.118^*^	14.140	0.000
	Process	−86.956^*^	14.132	0.000

*The mean difference is significant at the 0.05 level.

Dependent Variable: Forgive post-test.

The results presented showed a significant difference in the forgiveness level of the participants in the REACH group and the control group (*p < 0*.001). Likewise, a statistically significant difference was found in the levels of forgiveness for participants in the Process group and the control group (*p* < 0.001). The result further revealed that there was no significant difference in the forgiveness level of participants in the two experimental groups (REACH group and Process group) (*p* = 0.999). The results imply that both the REACH Forgiveness Model and the Enright Process Model are effective interventions for increasing forgiveness among participants compared to no intervention. However, since no significant difference was found between the two intervention groups, it suggests that both models are equally effective and can be used interchangeably in counseling practice. The adjusted mean scores of the participants within the various groups are presented in Table 5.

**Table 5:** Estimated marginal means.

Groups	Mean	Standard deviation
REACH	253.86	10.13
Process	252.69	10.31
Control	165.74	9.76

*Hypothesis 2:* There will be a statistically significant difference in forgiveness scores between participants exposed to the intervention models and those in the control group after controlling for pre-test scores.

To further examine differences among the groups, the estimated marginal means (adjusted means) of the post-test forgiveness scores were analyzed after controlling for pre-test scores. The results are presented in Table 5.

The descriptive statistics showed that after controlling for the pre-test scores on forgiveness, the mean score for the participants in the REACH group (*M =* 253.86, *SD =* 10.13) was significantly higher than the mean score of participants in the control group (*M* = 165.71, *SD* = 9.76). Similarly, the mean score for the participants in the Process group (*M* = 252.69, *SD* = 10.31) was also significantly higher than the mean score of participants in the control group (*M =* 165.71, *SD =* 9.76), after controlling for the pre-test scores on forgiveness. Though there was a slight difference in the marginal mean scores between participants in the REACH group (*M =* 253.86, *SD =* 10.13) and the Process group (*M =* 252.69, *SD* = 10.31), the difference was not significant.

On the whole, the outcome of the analysis showed that both the REACH model and the Process model were effective in enhancing forgiveness among College of Education students. Participants who were exposed to the two models (Process and REACH) exhibited a significant improvement in forgiveness levels. These participants were able to forgive the people who hurt them. Although both therapies were found to be effective in reducing students’ unforgiveness, neither therapy was found to be more effective than the other. In other words, the REACH model did not demonstrate a higher level of effectiveness in improving forgiveness compared to the Process model, and vice versa.

Integrating the results, the qualitative findings were introduced to assess how the treatments worked and to identify any variations in the results. Specifically, the qualitative results were compared with the quantitative findings.

### Post-intervention responses

This section presents the themes that emerged from participants’ interviews after the intervention. The themes included positive feelings toward the wrongdoer, positive thoughts about the wrongdoer, and positive behavior toward the offender.

#### Positive feeling toward offender

The responses of the participants revealed that they had more positive feeling toward the offender. They expressed that their feelings about the offender had changed and that they were willing to speak to the offender. Below are some of the comments participants shared with the researcher:

*“I no longer feel angry [sic] towards her after the intervention. I have even called her recently. After going through all the lessons, I now have compassion her than before”*. (Participant 1)

*“How I feel towards her has changed, right now the feeling is more positive than negative.”* (Participant 2)

*“I am okay because I don’t think about what happened and feel hurt anymore.”* (Participant 3)

*“My feeling towards my brother is not like before where the thought of him gets me angry.”* (Participant 4)

#### Positive thought about offender

Almost all the participants described their thoughts about the offender as positive after the intervention.

*“Right now, my thoughts about the person are more positive and I am also trying to see if I can help her so that she will stop that kind of behavior”*. (Participant 2)

*“I don’t have any bad thought about the person unlike before.”* (Participant 4)

*“Now I wish her well so I don’t have any negative thought about the person.”* (Participant 1)

#### Positive behavior toward offender

The participants shared their views regarding how they would behave toward the persons who hurt them after the intervention. They indicated that they would react positively to those individuals, likely because they felt enlightened after the intervention. They shared the following views:

*“I will talk to the person. Thus, I will greet him and ask how he is doing. This I will not do initially when he offended me.”* (Participant 2)

*“I will exchange greetings with her and respond well to her unlike before.”* (Participant 3)

*“I will greet them. I will talk to them relate well with them”*. (Participant 1)

*“I will greet him and talk to him.”* (Participant 4)

#### Influence of the intervention

The participants spoke about how their interaction with the research team has influenced their perception of not holding on to offenses and forgiving the offender. The participants explained that they have come to realize that there is no need to withhold forgiveness from an individual who has offended them.

*“I have learned that hurt can destroy my life so I have to let go of the past based on the lessons.”* (Participant 1)

*“…because of what I have learned from our interactions I don’t think it is even necessary to hold on to that hurt.”* f*rom what I learned, there is no need to hold grudge against the person who offended me so I have let go of everything.”* (Participant 2)

*“…because of the lessons I went through I don’t want to hurt myself so I will say I have forgiven them.”* (Participant 3)

*“I will give him a gap. Though I don’t have anything against him.”* (Participant 4)

#### Relational recalibration (forgiveness with boundaries)

Participants also distinguished forgiveness from reconciliation. One student shared,

“I can forgive her in my heart, but I don’t trust her with my secrets again.”

Another noted,

*“We greet now, but I avoid being alone with him.”* (Participant 1)

These narratives show that forgiveness was not simply about renewed closeness but about negotiating safety and dignity in relationships.

#### Ambivalence and relapse

Forgiveness was not linear; some hesitated between empathy and resentment. A participant admitted,

*“When I see her, I remember the pain, and sometimes the anger comes back, even though I prayed to forgive.”* (Participant 3)

This suggests that forgiveness is a process marked by setbacks, not a one-time achievement.

#### Agency and meaning-making

Participants reported personal growth and identity shifts. One reflected,

*“Now, when I have conflict with my roommate, I try to calm myself before reacting. I never thought I could control my temper like this.”* (Participant 2)

Another connected the intervention to faith:

“*The teaching reminded me of my church sermons, but now I see it is also about my own peace of mind, not just doing what others expect.”* (Participant 4)

The qualitative findings provide deeper insight into how the interventions influenced participants’ forgiveness experiences and help explain the quantitative results. Participants’ statements showed clear changes in their emotional responses, thoughts, and behaviors toward offenders, indicating that the interventions facilitated the development of positive feelings, compassionate thinking, and constructive interactions with those who had hurt them. For example, participants reported reduced anger, increased willingness to communicate with offenders, and the ability to wish them well, demonstrating the transformation from negative to positive affect, cognition, and behavior—key indicators of forgiveness. The responses also revealed that participants understood forgiveness as a process involving reflection, emotional regulation, and personal growth. Importantly, some participants distinguished forgiveness from reconciliation by setting boundaries in their relationships, suggesting that the interventions helped them pursue forgiveness while maintaining personal safety and dignity. Participants also acknowledged that forgiveness was not always linear, as occasional feelings of anger resurfaced, highlighting the ongoing and complex nature of the forgiveness process. Overall, these narratives illustrate how the REACH Forgiveness Model and the Enright Process Model helped participants reinterpret interpersonal hurt, regulate emotional reactions, and develop healthier relational attitudes, thereby supporting the quantitative findings that both interventions significantly improved forgiveness levels among College of Education students. In summary, the analysis indicated that both the REACH model and the Enright Process Model were effective in increasing forgiveness among College of Education students. The qualitative results align with the quantitative results, revealing that participants demonstrated a significant improvement in their levels of forgiveness. Specifically, these participants were able to forgive those who hurt them by developing positive affect, cognition, and behavior toward the offender.

## Discussion

*Hypothesis 1:* Participants who receive the Enright Process Model and REACH Forgiveness Model intervention will demonstrate significantly higher forgiveness scores at post-test compared to participants in the control group.

The current study’s findings confirmed Hypothesis 1, which predicted that participants who received the Enright Process Model and REACH Forgiveness Model interventions would be more forgiving than those in the control group. The findings revealed that participants exposed to the two intervention models demonstrated a statistically significant improvement in forgiveness scores at the post-test compared to those in the control group. This suggests that structured forgiveness programs can significantly enhance forgiveness among College of Education students. This study found that the Enright Process Model and the REACH Forgiveness Model were effective, which aligns with earlier empirical findings. For example, López et al. (2021), Kurniati et al. (2020) and Fayyaz and Besharat, 2011 found that forgiveness interventions based on these models consistently reduced unforgiveness while increasing forgiveness and empathy among people who had experienced interpersonal trauma. Similarly, Reed and Enright (2006) and Landry et al., 2005 discovered that forgiveness treatment allows victims of injustice to actively choose forgiveness as a constructive response to interpersonal offenses. These data support the assertion that forgiveness programs can change people’s emotional, cognitive, and behavioral responses to offenders. The findings are also consistent with the theoretical perspectives of Worthington (2020), Worthington et al. (2019), Baskin and Enright, 2004 and Enright (2001), who emphasize that forgiveness processes require the development of empathy, compassion, humility, and understanding for offenders. In the current study, participants who received the intervention reported a decrease in negative emotions such as anger, vengefulness, and resentment, while also gaining positive feelings such as empathy, sympathy, and compassion for those who had hurt them. These results demonstrate how forgiveness therapies can promote emotional healing and reduce interpersonal conflict.

The qualitative findings corroborated the quantitative results by illustrating how participants experienced the forgiveness process during the intervention. Many participants described how structured reflection activities helped them reassess their attitudes toward the perpetrator and acknowledge the emotional toll of holding onto resentment. For example, one REACH group participant stated, “When I thought of how my friend also grew up without support, I felt less angry at her insult.” This response highlights the importance of empathy development, a key component of the REACH forgiveness approach. Such experiences indicate that the intervention helped participants reinterpret the offender’s behavior and lessen negative emotional responses. Overall, the data suggest that forgiveness interventions can help people overcome interpersonal hurt and restoring emotional wellbeing. The findings confirm that both the Enright Process Model and the REACH Forgiveness Model are effective therapeutic tools for fostering forgiveness among college students.

*Hypothesis 2:* There will be a statistically significant difference in forgiveness scores between participants exposed to the intervention models and those in the control group after controlling for pre-test scores.

The findings also supported Hypothesis 2, which anticipated a statistically significant difference in forgiveness ratings between participants exposed to the intervention models and those in the control group after accounting for pre-test scores. The ANCOVA results revealed that, even after considering initial differences in pre-test forgiveness levels, individuals in both intervention groups had significantly higher post-test forgiveness scores than those in the control group. This finding suggests that the observed gains in forgiveness were due to the intervention rather than pre-existing disparities among individuals. Interestingly, while both intervention groups demonstrated significant increases in forgiveness, there was no statistically significant difference between the Enright Process Model and the REACH Forgiveness Model. This indicates that both intervention modalities are equally effective at encouraging forgiveness. This finding is consistent with earlier research showing that both models are empirically supported and frequently employed in forgiveness programs (Osei-Tutu et al., 2020; Worthington, 2020; Toussaint et al., 2020; López et al., 2021; Rahman et al., 2018). The lack of significant differences between the two models suggests that they may achieve similar results through distinct psychological mechanisms.

The qualitative findings provided more insight into how the two concepts influenced participants’ forgiving experiences. Participants in the REACH intervention typically reported feelings of emotional relief and empathy for the perpetrator, aligning with the model’s emphasis on empathy development and altruistic forgiveness. In contrast, participants in the Enright Process Model group frequently cited cognitive reflection and moral decision-making as critical components of their forgiveness journey. According to one participant, “I told myself forgiveness is the right thing, even if I still felt hurt.” This statement highlights the Enright model’s emphasis on forgiveness as a deliberate moral decision based on personal beliefs. These qualitative variations indicate that, while the two models promote forgiving outcomes in similar ways, they may follow slightly distinct psychological paths. The REACH approach appears to foster forgiveness through emotional transformation and empathy development, whereas the Enright Process approach focuses on cognitive restructuring and a moral commitment to forgiveness.

Culturally, the findings highlight the importance of forgiveness interventions in the Ghanaian setting. Participants frequently described forgiveness as a multifaceted process impacted by cultural values, social expectations, and personal safety concerns. Forgiveness was often viewed as an internal process of releasing anger while preserving proper relational boundaries, rather than as final reconciliation. This perspective is consistent with the overall emphasis of Ghanaian society on peace and interpersonal relationships. Despite the positive outcomes of the current study, the findings differ from those of Evensen (2013) and Neto et al., 2007, who suggested that forgiveness is not always the best response to injustice, especially when victims cannot directly relate to the offender. According to Evensen, some people may consider certain actions unforgivable due to the magnitude of the harm caused. Nonetheless, the current findings indicate that structured therapeutic interventions can help people process interpersonal harm and develop healthy emotional responses. Overall, the information provided shows that both the Enright Process Model and the REACH Forgiveness Model are effective ways to promote forgiveness among college students. The findings emphasize the value of structured therapeutic processes in assisting individuals to reflect on the consequences of unforgiveness and to cultivate empathy, compassion, and understanding for offenders.

## Conclusion

This study examined the effects of the Enright Process Model and the REACH Forgiveness Model on promoting forgiveness among students in Colleges of Education in the Ashanti Region of Ghana. The findings demonstrate that both intervention models significantly increased participants’ levels of forgiveness following experiences of interpersonal hurt. Students who participated in either structured forgiveness intervention reported higher post-test forgiveness scores compared to those in the control group, indicating that guided forgiveness programs can effectively reduce negative emotional responses such as anger, resentment, and revenge while fostering positive responses such as empathy, compassion, and understanding. After controlling for pre-test scores, the analysis further revealed that the improvement in forgiveness levels was primarily attributable to the intervention programs rather than to pre-existing differences among participants. However, the results showed no significant difference in effectiveness between the Enright Process Model and the REACH Forgiveness Model, suggesting that both models are equally effective in facilitating forgiveness among college students.

The qualitative findings reinforced these results by showing that participants experienced forgiveness as a gradual and reflective process involving emotional processing, cognitive reframing, empathy development, and intentional decision-making. By demonstrating the effectiveness of two established forgiveness intervention models within the context of Ghanaian Colleges of Education, this study contributes to the existing literature by providing empirical evidence that structured forgiveness interventions can be successfully applied in higher education settings in Ghana. The findings extend the body of knowledge on forgiveness interventions by confirming their relevance and applicability in non-Western educational contexts and highlight their potential as practical tools for promoting emotional wellbeing and healthy interpersonal relationships among college students.

## Recommendations

Based on the findings of this study, several practical implementations are recommended at the individual, institutional, policy, and research levels. At the individual level, students should be encouraged to participate in structured forgiveness training programs to help them effectively manage interpersonal hurt, regulate negative emotions, and develop empathy and healthy coping strategies. At the institutional level, counselors in Colleges of Education should integrate evidence-based forgiveness interventions, such as the Enright Process Model and the REACH Forgiveness Model, into their counseling and student support services. Institutions may also organize workshops, seminars, and psychoeducational programs that promote forgiveness, emotional regulation, and conflict resolution among students. At the policy level, educational authorities and college administrations should consider incorporating forgiveness education and emotional wellbeing programs into student development initiatives and counseling policies to foster healthier interpersonal relationships and supportive campus environments. Finally, for future research, scholars should examine the long-term effects of forgiveness interventions, explore their applicability in other educational levels and cultural contexts, and investigate additional psychological and social outcomes associated with forgiveness interventions to further strengthen the evidence base in this field.

## Data Availability

The raw data supporting the conclusions of this article will be made available by the authors, without undue reservation.
